# Implicit Versus Explicit Local Contextual Processing

**DOI:** 10.1371/journal.pone.0065914

**Published:** 2013-06-13

**Authors:** Noa Fogelson, Miguel Fernandez-del-Olmo

**Affiliations:** 1 Department of Psychology, University of A Coruña, La Coruña, Spain; 2 Department of Physical Education, University of A Coruña, La Coruña, Spain; University of California, Davis, United States of America

## Abstract

We investigated the effects of implicit local contextual processing using behavioral and electrophysiological measures. EEG recording blocks consisted of targets preceded by either randomized sequences of standards or by sequences including a predictive sequence signaling the occurrence of a target event. Subjects performed two sessions: in the first the regularity of the predictive sequence was implicit, while in the second this regularity was made explicit. Subjects pressed a button in response to targets. Both the implicit and explicit sessions showed shorter reaction times and peak P3b latencies for predicted versus random targets, although to a greater extent in the explicit session. In both sessions the middle and last most-informative stimuli of the three-standard predictive sequence induced a significant larger P3b compared with randomized standards. The findings show that local contextual information is processed implicitly, but that this modulation was significantly greater when subjects were explicitly instructed to attend to target-predictive contextual information. The findings suggest that top-down attentional networks have a role in modulating the extent to which contextual information is utilized.

## Introduction

Implicit learning is a non-conscious process by which task performance is facilitated, without the verbalized awareness of the subject. The functional significance of implicit learning is to increase processing of information to a greater extent than can be achieved through conscious functions alone [1], [2]. Implicit learning is thought to engage cognitive resources and processes such as working memory [1], [3]. There is evidence that cortical and subcortical areas such as the prefrontal cortex, parietal cortex, medial temporal lobe and basal ganglia [4], [5], [6], [7], [8], [9] are involved in implicit learning. Recent studies have suggested that it is not one region but rather differential networks which have a role in determining whether information is processed implicitly or explicitly [10], [11]. Several experimental paradigms have been used to study implicit learning, including implicit motor sequence learning [12] and contextual cueing [2] where associations between spatial configuration and target location are learned in an incidental manner. It remains inconclusive whether implicit learning and explicit learning are overlapping processes [3], [13], [14] or whether these functions have distinct neural substrates [15], [16], [17].

In the present study we investigated the ability to detect and utilize predictive local contextual information to predict target events in an implicit manner compared to when it is processed explicitly [18], [19].

Contextual processing is a specific subcomponent of working memory that enables extraction of relevant information from our environment in order to facilitate the selection of appropriate task-specific responses [20], [21], [22]. The processing of context may be measured using the P3b component which has been suggested to be a measure of the evaluation of environmental signals [23], [24], [25], [26], [27] and of monitoring processes mediating perceptual analysis and response initiation [27]. The P3b, is elicited, among other tasks, by targets in the classical oddball target detection task [28]. P3b amplitude has been shown to increase with increasing stimulus value or relevance to the task [29], [30] and is affected by stimulus salience and allocation of attention to the stimulus [30]. Studies of implicit learning have shown reduced P3b amplitudes for target or deviant stimuli in implicit learning compared with explicit learning [15], [31], [32]. Others have demonstrated that negative event-related activity around 300ms can determine whether subjects became aware of visual patterns in an inattentional blindness paradigm [33], [34].

Previous studies have shown that explicit processing of predictive local contextual information, defined as the occurrence of a short predictive series of visual stimuli before the appearance of a target event, facilitates target detection [18], [19], [35]. These studies identified several neural correlates associated with the facilitation of explicit local contextual processing. First, P3b latency and reaction time was faster for the processing of predicted compared with random targets. Second, there was a gradual increase in P3b amplitude during the detection of the stimuli consisting of the predicting sequence, indicating that the predictive sequence became a secondary target for the subjects. In the present study we employed this paradigm [18], [19] to investigate the effects of implicit versus explicit local contextual processing. The objective of the study was to determine whether the facilitatory effect of explicit processing of predictive local context [18], [35] is also demonstrated when the same stimuli are processed implicitly. To this end, subjects performed two sessions of the same task. Both sessions were identical and consisted of visual stimuli of triangles facing either left, up, right or down [18], [19]. In the first session subjects are simply instructed to detect the target (downward facing triangle), while in the second session they are made aware that targets either appear randomly or after a predictive sequence (triangles facing left, upward, and then right) that signals the occurrence of predictive targets. This design allows for the direct comparison of behavioral and electrophysiological measures between the implicit and the explicit session in the same subjects, thus avoiding confounds of inter-individual variability [33], [34]. Thus, we expected to replicate behavioral and electrophysiological results [18], [19] for the explicit session and to determine whether there are local context effects in the implicit session. Our hypothesis was that if local predictive information is processed implicitly, we would observe similar behavioral and electrophysiological indices identified for explicit local contextual processing.

## Methods

### Participants

12 subjects participated in the study (mean age ± standard deviation = 22.7±3.4 years, 1 female). Subjects were right handed, had normal vision and had no history of psychiatric or neurological problems. The Ethics committee of University of A Coruña approved the study. A written consent was obtained from all the subjects.

### Task

**Figure 1:**
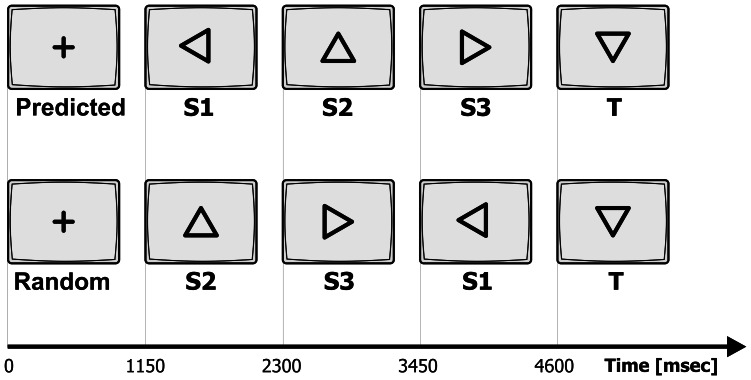
Task timeline. Stimuli presented in the sessions. Sequences of standards S1, S2 and S3 with a predicted sequence (top) and in randomized order (bottom) preceding the target (T). The predictive sequence is always S1 followed by S2 and then S3 (n-1). Inter-trial intervals, including duration of stimulus presentation (150 ms) are displayed. Each block consisted of 6 different randomized sequences of standards (3–8 standards long) preceding the target; and 6 sequences of standards (3–8 standards long) with the predictive sequence preceding the target in each.

Subjects sat 110 cm in-front of a 21-inch PC-computer screen. Stimuli were presented in the center of the visual field. Subjects were asked to centrally fixate throughout the recording. Stimuli consisted of 15% targets and 85% of equal amounts of three types of standards. In each block a total of 78 stimuli (12 targets, 22 of each standard type) were presented each for 150 ms and inter-stimulus interval (ISI) of 1 second. Recording blocks consisted of targets preceded by either randomized sequences of standards or by sequences including a three-standard predictive sequence. The target was a downward facing triangle and the three standards were triangles facing left, upwards and right, at 90 degree increments. The predictive sequence always consisted of the three standards of triangles facing left, up and right, always in that order. Figure 1 illustrates an example of a target preceded by a randomized sequence of standards and a target preceded by the predictive sequence of standards for each of the sessions. The predictive sequence was always followed by a target. Each block consisted of 6 different randomized sequences of standards (3–8 standards long) preceding the target; and 6 sequences of standards (3–8 standards long) with a predictive sequence preceding the target in each. Each recording session consisted of 10 different blocks, displayed in randomized order, each approximately 1.6 minutes long.

Two sessions were performed by each subject: an implicit session and then an explicit session. Before the first (implicit) session, subjects were told that a series of triangles would be presented on the screen and were instructed to respond by pressing a button, using their right index finger, whenever they detected a downward facing triangle. Subjects performed a brief training session to ensure they were able to detect the target accurately before the recording session began. After completing the first recording session subjects were questioned whether they noticed at any point during the task that they were able to anticipate the occurrence of the downward facing triangle, i.e the target. Subsequently subjects were then shown the predictive sequence and were told that it would be 100% predictive of a target, but that targets would also appear randomly throughout the block. Subjects were asked to press a button each time a target was presented, to pay attention and look for the predictive sequence, and to avoid premature responses. Subjects then performed a brief training session to ensure that they were confident in the detection of the predictive sequence, before the second recording session began. Stimulus presentation and response recordings were controlled using E-prime (Psychology Software Tools, Inc., Pittsburgh, USA).

### EEG Recordings

EEG was recorded from a 64 Ag-AgCl electrode array using the ActiveTwo system (Biosemi, The Netherlands). Signals were amplified and digitized at 512 Hz. Post processing and ERP analysis of the data was performed using Brain Vision Analyzer (Brain Products GmbH, Germany). All channels were re-referenced to averaged linked earlobes.

### Behavioral Analysis

Accuracy was defined as the percentage of targets for which a button press was detected.

Reaction times were calculated by averaging correct trials for predicted and random targets in each subject for each session. Misses (no button press 150–1150 ms post-stimulus onset) were excluded from reaction time analysis. Premature responses were not taken into consideration in the analysis of reaction time. Reaction times were analyzed using E-prime (Psychology Software Tools, Inc., Pittsburgh, USA).

### ERP Analysis

Prior to ERP analysis blinks were defined using ICA (64 EEG electrodes were included), and the component identified as a blink was removed using the linear derivation function in Brain Vision Analyzer. Epochs containing premature responses, misses (no button press 150–1150 ms post-stimulus onset) and eye saccades were excluded from further analysis. EEG signals were filtered at 0.1–30 Hz for subsequent analysis. EEG signals were sorted and averaged relative to the stimulus onset, with epochs set from −200 to 1000 ms relative to stimulus onset. EEG epochs with amplitude of more than 75 µV at any electrode were excluded.

### N1

To assess the early perceptual processes between the two target conditions, peak N1 amplitudes (measured in µV) were determined at PO7 and PO8, for both predicted and random targets presented. N1 was determined as the most negative peak in the latency range of 50–200 ms. Comparable effects were observed for both electrodes and since stimuli were presented centrally, we utilized data from PO7 in statistical analysis of N1 in order to restrict the number of comparisons.

### P3b

P3b was determined as the most positive point in the latency range of 300–700 ms. In order to restrict the number of comparisons an omnibus ANOVA was first performed. In this ANOVA we used the P3b peak amplitude at electrode sites AFz, Fz, FCz, Cz, CPz, and Pz for six different conditions (predicted and random targets, standards and the three standards comprising the predicting sequence) across both sessions (implicit and explicit). We found that across sessions maximal P3b amplitudes were observed at electrode site CPz. Thus, for each subject peak P3b amplitudes (measured in µV) at CPz were evaluated for 6 conditions in each session: targets after predictive sequences (predicted), targets after non predictive random sequences (random), random preceding standards (standards excluding those comprising the predicting sequence) and the three standards consisting of the predictive sequence: the last most-informative standard (n-1), the middle standard (n-2) and the first least-informative standard (n-3) of the predicting sequence. We also evaluated P3 amplitude at CPz for each of the three standards presented randomly, that is, not as a part of the predictive sequence (Sn-1, Sn-2 and Sn-3) in order to compare each of these standards to its counterpart when presented within the predictive sequence. In addition, we evaluated P3b amplitudes at CPz for conditions n-1, n-2, n-3, Sn-1, Sn-2 and Sn-3, during the first 5 and last 5 blocks of the implicit session, to determine whether implicit processing of the predictive sequence changed as a function of time (i.e. between the first half and last half of the implicit session).

Peak P3b latencies (measured in ms) were evaluated for predicted and random targets at the electrode site with the largest P3b amplitude.

### Statistical Analysis

Analysis of variance (ANOVA) was performed with the Greenhouse-Geisser correction, followed by post-hoc parametric paired t-tests, Sidak corrected for multiple comparisons unless otherwise stated. Mean values with ± standard error of the mean (SEM) are used throughout the text. Partial eta squared (η_p_^2^) values are reported where applicable. Pearson’s Product Moment correlation coefficient was used to calculate correlations.

## Results

### Behavioral Results

After the implicit session all subjects reported that they did not notice at any point during the task that they were able to anticipate the occurrence of a target, and were not aware of the presence of a predictive sequence.

To test whether accuracy of target detection was comparable across sessions we performed an ANOVA for accuracy with condition (predicted, random targets) and session (implicit, explicit) as the repeated measures factors. There was no significant main effect for condition (F(1,11) = 1.80, p = .206, η_p_^2^ = .14) or session (F(1,11) = .53, p = .480, η_p_^2^ = .05). Overall mean accuracies for predicted targets were 99.2±.4% and 98.5±.7% and for random targets 99.3±.2% and 99.3±.4% for the implicit and explicit sessions, respectively. There were no significant differences in accuracy between the two sessions.

**Figure 2:**
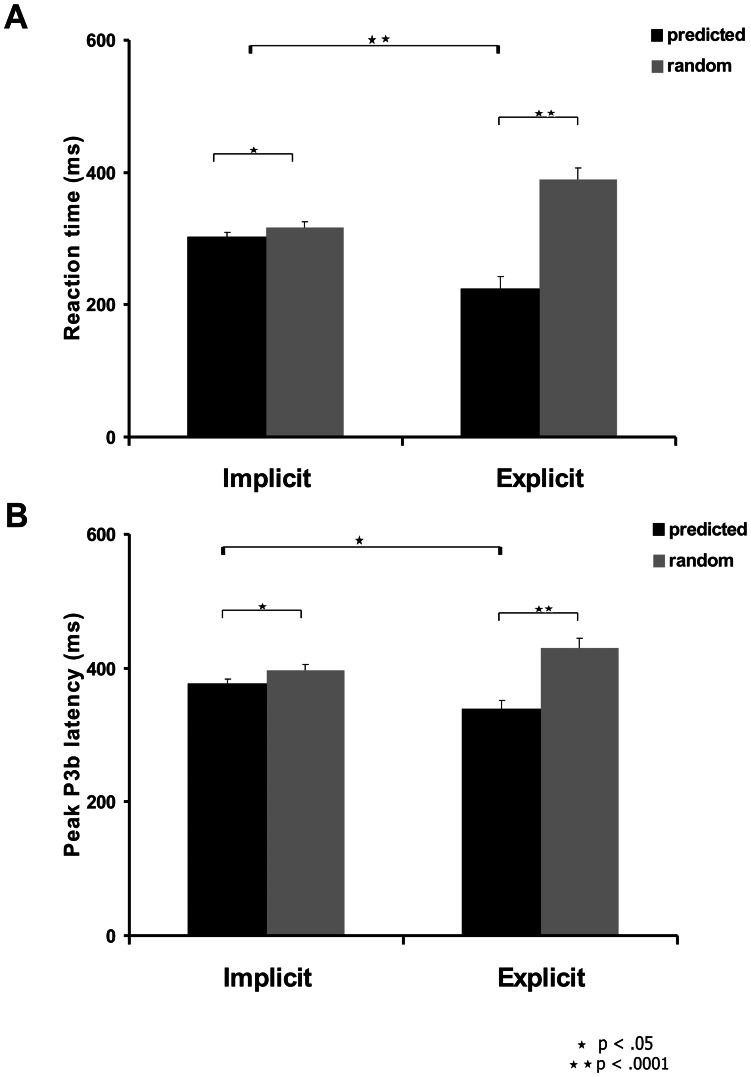
Reaction times (A) and P3b peak latency at CPz (B) for predicted and random targets in the implicit and explicit sessions. Bars = SEM.

To compare the reaction times (RT) for the targets and to test whether there is a behavioral facilitation in the implicit and explicit sessions, we performed an ANOVA with condition (predicted, random targets) and session (implicit, explicit) as the repeated measures factors. There was a main effect for condition (F(1,11) = 154.95, p<.0001, η_p_^2^ = .93) but no main effect for session (F(1,11) = .05, p = .828, η_p_^2^ = .004). However, there was a significant condition×session interaction (F(1,11) = 70.94, p<.0001, η_p_^2^ = .166). In the implicit session, RTs for predicted targets (mean RT = 302±8 ms) were faster than RTs for random targets (mean RT = 317±8 ms, t(11) = 3.9, p = .002). In the explicit session RTs for predicted targets (mean RT = 224±18 ms) were also faster than RTs for random targets (mean RT = 389±18 ms, t(11) = 10.5, p<.0001). RTs for predicted targets were shorter in the explicit compared with the implicit session (t(11) = 5.9, p<.0001), while RTs for random targets were longer in the explicit compared with the implicit session (t(11) = 4.2, p = .001). RT comparisons are displayed in Figure 2a.

### N1

We utilized an ANOVA with condition (predicted, random targets) and session (implicit, explicit) as repeated measures factors, to compare the peak N1 amplitude at electrode site PO7, between predicted and random targets. There was no significant main effect for condition (F(1,11) = .30, p = .596, η_p_^2^ = .03) or session (F(1,11) = 1.71, p = .218, η_p_^2^ = .13), and no significant interaction (F(1,11) = .35, p = .57, η_p_^2^ = .03).

### P3b Amplitude

**Figure 3:**
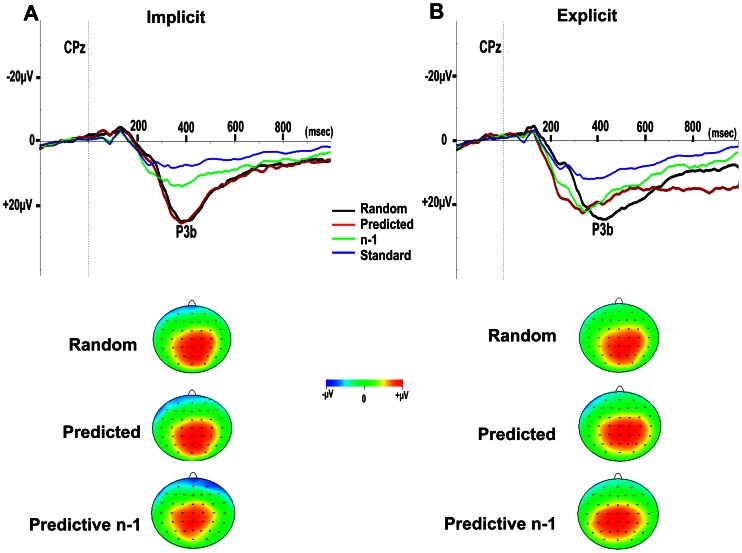
Grand average at CPz for the 4 conditions: targets after random non predictive (Random) and predictive sequences (Predicted), the last most informative standard comprising the predicting sequence (n-1) and random preceding standards (Standard); and the topographic maps of predicted, random targets and n-1, for implicit (A) and explicit (B) sessions. Vertical dotted lines indicate time of stimulus presentation onset.

**Figure 4:**
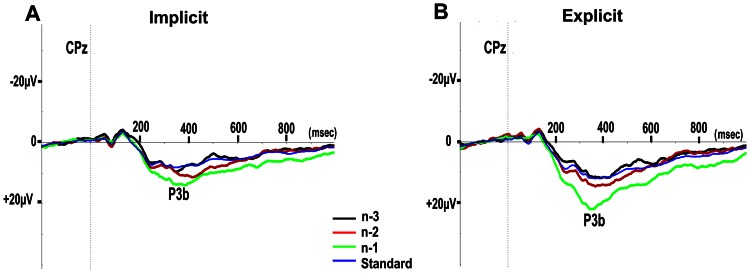
Grand average at CPz for the three stimuli comprising the predictive sequence. The first least-informative (n-3), the middle (n-2) and last most informative (n-1); and for random preceding standards (Standard) for implicit (A) and explicit (B) sessions. Vertical dotted lines indicate time of stimulus presentation onset.

Waveforms of the grand-averaged ERPs across the 12 subjects, at electrode site CPz elicited by predicted and random targets, standards and the three standards of the predictive sequence (n-3, n-2, and n-1, the last most-informative stimulus of the predicting sequence), for implicit and explicit sessions are shown in Figure 3 and Figure 4.

To test whether the targets and the last most informative stimulus of the predictive sequence (n-1) induced significantly larger P3b amplitudes compared with randomized standards across the sessions, we compared peak P3b amplitudes at electrode site CPz and performed an ANOVA with condition (predicted, random targets, n-1, standards), and session (implicit, explicit) as the repeated measures factors. There was a significant main effect for condition (F(3,33) = 31.55, p<.0001, η_p_^2^ = .74, epsilon = .68), but no significant main effect for session (F(1, 11) = 1.34, p = .271, η_p_^2^ = .11). However, there was a significant condition×session interaction (F(3,33) = 9.82, p<.0001, η_p_^2^ = .47, epsilon = .76).

In the implicit session, post-hoc tests corrected for multiple comparisons, showed that peak P3b amplitudes were larger for predicted targets (mean peak P3b amplitude = 27.8±2.0 µV) and random targets (mean peak P3b amplitude = 27.7±2.1 µV) compared with n-1 (mean peak P3b amplitude = 17.3±2.0 µV, p≤.02), and standards (mean peak P3b amplitude = 10.8±1.1 µV, p<.0001). Importantly, P3b amplitudes were also larger for n-1 (p = 0.019) compared with standards.

In the explicit session, post-hoc tests corrected for multiple comparisons, showed that peak P3b amplitudes were larger for predicted targets (mean peak P3b amplitude = 23.8±1.7 µV), random targets (mean peak P3b amplitude = 26.6±2.0 µV), n-1 (mean peak P3b amplitude = 23.4±2.5 µV) compared with standards (mean peak P3b amplitude = 14.8±1.4 µV, p≤.006).

In both sessions there was no significant difference in P3b amplitude between random and predicted target stimuli. In addition, P3b amplitudes for the n-1 (t(11) = 2.6, p = .027), and standard (t(11) = 5.3, p<.0001) conditions were significantly larger in the explicit compared with the implicit session.

**Figure 5:**
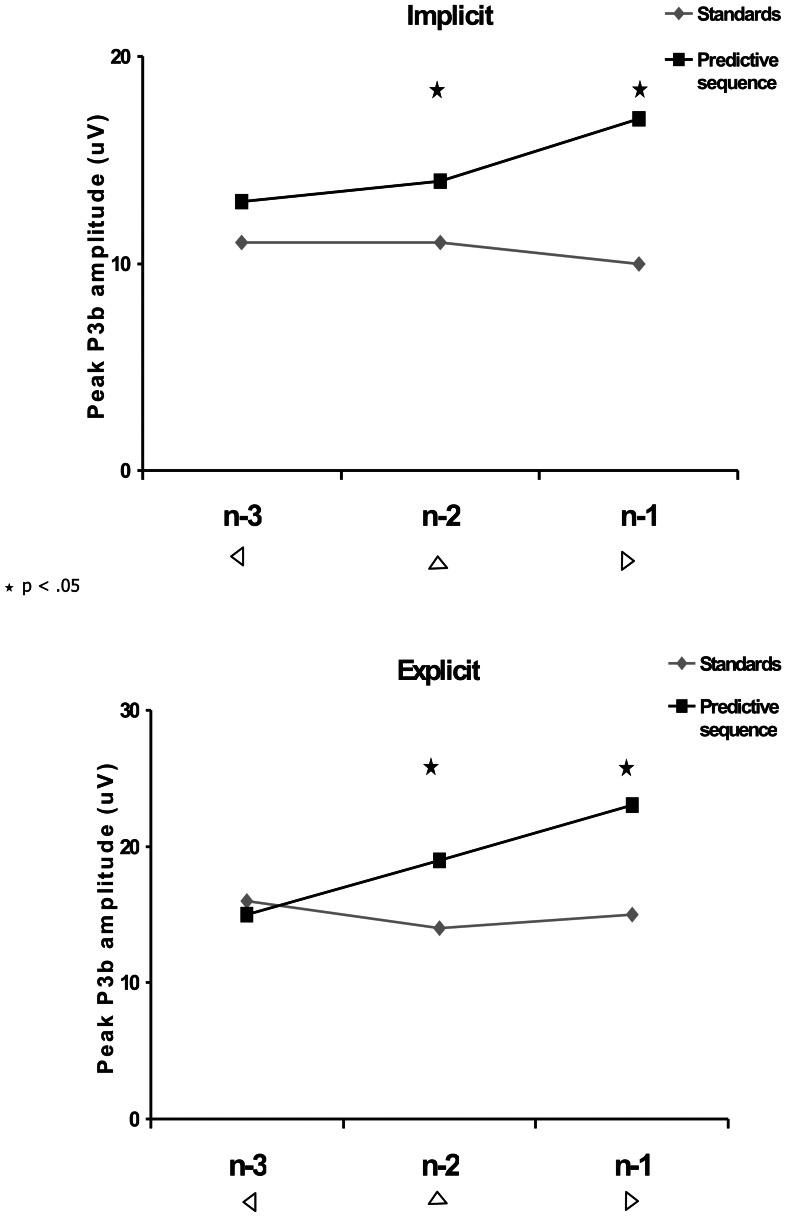
P3b amplitudes at CPz for the three stimuli comprising the predictive sequence. The first least-informative (n-3), the middle (n-2), and last most informative (n-1); and for the corresponding three standards presented randomly (Standards) for implicit and explicit sessions.

To test whether the predictive sequence induced significantly larger P3b amplitudes compared with the same stimuli presented in a randomized order across the two sessions, we compared peak P3b amplitudes at electrode site CPz of each of the predictive standards n-1, n-2 and n-3 and each of these standards presented randomly (Sn-1, Sn-2, Sn-3). An ANOVA was performed with session (implicit, explicit), prediction (predictive, randomized), and stimulus (triangle facing left, upwards, right) as the repeated measures factors. There was a significant main effect for session (F(1,11) = 16.5, p = .002, η_p_^2^ = .60), showing larger P3b amplitudes in the explicit session than in the implicit session. In addition there was a significant main effect for prediction (F(1,11) = 57.26, p<.0001, η_p_^2^ = .84) and stimulus (F(2,22) = 10.46, p = .001, η_p_^2^ = .49, epsilon = .87), and a significant stimulus×prediction interaction (F(2,22) = 17.35, p<.0001, η_p_^2^ = .61, epsilon = .80), but no significant interaction of stimulus×prediction×session (F(2,22) = 2.38, p = .119, η_p_^2^ = .18). Post-hoc t-tests showed that in both the implicit and explicit session P3b amplitudes in n-1 were larger compared to Sn-1 (t(11) = 3.86, p = 0.003, and t(11) = 6.0, p<0.0001, in implicit and explicit session, respectively), n-2 was larger compared to Sn-2 (t(11) = 4.34, p = 0.001, and t(11) = 4.89, p<0.0001, in implicit and explicit session, respectively), and n-3 was not significantly different from Sn-3 (t(11) = 1.12, p = .286, and t(11) = 1.05, p = .315, in implicit and explicit session, respectively). Figure 5 demonstrates these comparisons.

To test whether the predictive sequence induced significantly larger P3b amplitudes compared with the same stimuli presented in a randomized order across the first 5 and last 5 blocks of the implicit session, we compared peak P3b amplitudes at electrode site CPz of conditions n-1, n-2, n-3 Sn-1, Sn-2, and Sn-3. An ANOVA was performed with block (first half, second half), prediction (predictive, randomized), and stimulus (triangle facing left, upwards, right) as the repeated measures factors. There was no significant main effect for block (F(1,11) = .236, p = .636, η_p_^2^ = .02) nor stimulus (F(2,22) = 2.38, p = .118, η_p_^2^ = .18, epsilon = .97). However, there was a significant main effect for prediction (F(1,11) = 19.14, p = .001, η_p_^2^ = .64), a significant stimulus×prediction interaction (F(2,22) = 6.75, p = .005, η_p_^2^ = .38, epsilon = .86), and a significant stimulus×prediction×block interaction (F(2,22) = 3.58, p = .048, η_p_^2^ = .25, epsilon = .82). Post-hoc t-tests showed a tendency for n-1 P3b amplitudes to be larger in the second half compared with the first half of the implicit session (t(11) = 2.14, p = 0.055).

### P3b Latency

To test whether processing speed of the two target conditions was modulated across sessions we compared peak P3b latencies at CPz and performed an ANOVA with condition (predicted, random targets) and session (implicit, explicit) as the repeated measures factors. There was a main effect for condition (F(1,11) = 32.6, p<.0001, η_p_^2^ = .75), but no main effect for session (F(1,11) = .03, p = .876, η_p_^2^ = .002). However, there was a significant condition×session interaction (F(1,11) = 27.0, p<.0001, η_p_^2^ = .71). Post hoc t-tests showed that in both implicit and explicit sessions peak P3b latency was shorter for predicted targets (mean P3b latency = 376±7 ms and 338±13 ms, for implicit and explicit sessions, respectively) compared with the peak P3b latency for random targets (mean P3b latency = 396±10 ms, t(11) = 2.7, p = .019 and 430±15 ms, t(11) = 6.0, p<.0001, for implicit and explicit sessions, respectively). Peak P3b latency for predicted targets were shorter in the explicit compared with the implicit session (t(11) = 2.6, p = .025), while P3b latency for random targets were longer in the explicit compared with the implicit session (t(11) = 2.6, p = .023). P3b latency comparisons are displayed in Fig. 2b.

### Correlations

In order to determine the association between the main behavioral and electrophysiological findings, behavioral measures were correlated with ERP measures across all the subjects in each session. We correlated RT of predicted targets, RT of random targets and the RT difference, with peak P3b latency for predicted and random targets, P3b latency shift, and n-1 peak P3b amplitude. In addition, to determine whether there was an association between the main ERP findings, we correlated n-1 peak P3b amplitude with peak P3b latency for predicted and random targets and the P3b latency shift. In the implicit session n-1 peak P3b amplitudes were correlated with the P3b latency shift (r = .609, p = .035). In the explicit session n-1 peak P3b amplitudes were correlated with RTs for predicted targets (r = -.755, p = .004).

## Discussion

We found similar effects of local contextual information across the implicit and explicit sessions. Neural correlates associated with local contextual processing [18], [19], [35] were identified in both the implicit and explicit session. First, in both sessions a significantly larger P3b was generated by predicted targets, random targets, compared with standards and by the middle, and last and most-informative stimuli of the predicting sequence, compared with the same standards presented in a randomized non-predictive sequence. Thus, in the explicit session the predictive sequence became a secondary target for the subjects and thus an indicator for successful local contextual processing, replicating results of earlier studies [18], [19], [35]. During the implicit session subjects reported that they were not aware of any predictive sequence. Nevertheless, during this session significantly larger P3b amplitudes were observed, particularly for the last most-informative stimulus of the predicting sequence (n-1), compared with randomized standards, suggesting that the sequence was detected implicitly. In addition, both sessions induced a gradual increase in P3b amplitude in the predictive sequence, supporting an accumulation of information from the preceding trials [18]. It has been suggested that implicit sequence learning depends on detection of variations in the environment through associative mechanisms [36]. Thus, one possible mechanism for the implicit detection of the predictive sequence may be that visual search processes are sensitive to regularities in the visual input [2]. This may be a likely mechanism since the sequence utilized a clockwise rotation, which is a familiar feature in our everyday surroundings. Another factor that may have contributed to P3b amplitudes increases is the alternating nature of the stimuli in the predictive sequence, while randomized standards also included repetitions [26], which may have confounded our conclusions. However, our findings argue against this possibility, showing positive correlations between n-1 P3b amplitudes and the target-evoked P3b latency shift during the implicit session, together with a tendency for n-1 P3b amplitudes to increase in the second half of the implicit session compared with the first half, suggesting implicit learning of the predictive sequence. Regardless of the mechanism by which the predictive sequence was detected, the predictive information seemed to have been processed implicitly as we will discuss below.

We found that in both the implicit and explicit sessions, reaction time and P3b latency were shorter for sequence predicted targets than for targets after non-predictive sequences, suggesting facilitation in the processing speed of these targets [18], [19], [37], [38], [39], [40], [41]. The fact that a significant reaction time difference and a P3b latency shift were observed between the two target conditions during the implicit session, suggests that subjects not only detected the predictive sequence in an implicit manner, but that the predictive information provided by this sequence was also utilized implicitly, in order to facilitate processing of deterministic targets, albeit to a significantly lesser extent compared with explicit contextual processing. This is further supported by correlations demonstrating that the larger the peak P3b for the predictive sequence preceding the target, the larger the target-evoked P3b latency shift that is observed in the implicit session. This is in line with other studies suggesting that useful visual predictive cues are encoded automatically in order to facilitate future stimulus interactions [2] and classification of objects [42], and that knowledge that is acquired in a non-conscious manner can automatically be used to facilitate performance [1].

In addition, there were no significant N1 amplitude differences between predicted and random targets (replicating Fogelson et al. [18]) in both sessions suggesting that perceptual processing was similar for predicted and random targets [43].

In summary, we found local context effects for both implicit and explicit processing. These findings suggest that local contextual processing can occur in an implicit as well as in an explicit fashion. We found that P3b ERP effects were modulated by both implicit and explicit local contextual processing, but that this modulation was significantly greater when subjects were explicitly instructed to attend to target-predictive contextual information.

### Modulation of Local Context in Explicit Versus Implicit Processing

The modulation observed in the present study during explicit contextual processing compared to implicit processing seems to be quantitative rather than qualitative, since a similar pattern of behavioral and electrophysiological indices of local context were observed in both the implicit and explicit sessions. These findings suggest that neural mechanisms underlying implicit and explicit local contextual processing overlap and may have a common source. The idea of overlapping processes for implicit and explicit processing is supported by other studies [3], [13], [14], although others have suggested that implicit learning may have distinct neural substrates from explicit learning [15], [16], [17]. There is evidence suggesting that the prefrontal cortex (specifically the dorsolateral prefrontal cortex) is critical for explicit local contextual processing [44], [45], [46] and that prefrontal top-down networks, such as fronto-striatal and fronto-parietal circuits, are also involved in this function [47], [48]. The frontal cortex has also been shown to be involved in implicit motor, sequence learning and contextual cueing [5,6,7,49], although other areas such as the parietal cortex, medial temporal lobe and basal ganglia have also been implicated [4], [8], [9], [16]. Others have shown that implicit learning of a hidden task structure involves a network connecting the hippocampus and medial prefrontal area, and when the structure of the task becomes explicit, there is a shift to activation of the frontal-parietal network [10].

The present study focuses on how local predictive context is processed implicitly. Our finding suggest that qualitatively similar neural processes may be involved in processing and utilizing predictive contextual information but that what is modulated is the magnitude to which context is used to facilitate detection of predictable targets. This modulation may occur through top-down attentional networks, so that increased attention during the explicit session, where subjects are instructed to detect and utilize the predictive sequence, facilitates processing of local contextual information compared to when it is processed implicitly. Our findings support this proposition, demonstrating larger P3b amplitudes during the processing of the predictive sequence compared to when these stimuli are processed implicitly. This is in line with studies implicating cognitive control systems as being important in guiding attention during explicit sequence learning [36], and of context-dependent modulation through attention driven top-down mechanisms [8,13,50]. In addition, since contextual processing is thought to be a specific subcomponent of working memory [20], [21], [46], our findings support evidence suggesting that working memory is involved in implicit learning [3], [36].

In conclusion, the findings of the current study suggest that predictive contextual information is processed both implicitly and explicitly, as indicated by both behavioral and electrophysiological correlates of local contextual processing, and that top-down attentional networks may have a role in modulating the extent to which local contextual information is utilized.
